# Predictive Value of Red Blood Cell Distribution Width for the Prognosis of Cardiac Arrest: A Systematic Review and Meta-Analysis

**DOI:** 10.31083/RCM43774

**Published:** 2025-11-17

**Authors:** Yufeng Zhang, Boyi Zhang, Maoxian Yang, Qianqian Wang, Peng Shen

**Affiliations:** ^1^Department of Medicine, Jiaxing University Master Degree Cultivation Base, Zhejiang Chinese Medical University, 314001 Jiaxing, Zhejiang, China; ^2^Intensive Care Unit, The First Hospital of Jiaxing, 314001 Jiaxing, Zhejiang, China; ^3^Affiliated Hospital of Jiaxing University, 314001 Jiaxing, Zhejiang, China

**Keywords:** cardiac arrest, red blood cell distribution width, patient outcome assessment, meta-analysis

## Abstract

**Background:**

While a potential relationship between red cell distribution width (RDW) and cardiac arrest (CA) prognosis has been raised, the question of whether there is robust data to support this connection remains open. To examine the association of red blood cell distribution width with prognosis in patients with cardiac arrest.

**Methods:**

This study strictly followed the Preferred Reporting Items for Systematic Reviews and Meta-Analyses (PRISMA) guidelines. Relevant studies were identified from searches conducted in the PubMed, Web of Science, the Cochrane Library, and Embase electronic databases up to March 12, 2025. The Newcastle–Ottawa scale was used to assess the quality of the included studies. The combined effect size was determined utilizing standardized mean differences (SMDs), hazard ratios (HRs), and 95% confidence intervals (CIs). Subgroup analyses were also performed to elucidate the sources of heterogeneity. Simultaneously, we also pooled sensitivity (SEN), specificity (SPE), diagnostic odds ratios (ORs), and the area under the summary receiver operating characteristic curve (AUROC).

**Results:**

This meta-analysis encompassed eight studies involving CA patients with CA. Our results suggested that patients who died after CA exhibited higher RDW levels than those who survived (SMD = 0.45; 95% CI: 0.30–0.60). There was a 1.63-fold higher risk of death in CA patients with high RDW levels versus those with low levels (95% CI: 1.27–2.08). The SEN, SPE, and AUC for using the RDW to predict mortality were 0.82 (95% CI: 0.74–0.88), 0.49 (95% CI: 0.23–0.74), and 0.80 (95% CI: 0.76–0.83), respectively.

**Conclusions:**

RDW is a relatively accurate predictor of prognosis in patients after CA. Thus, using RDW can provide some insights into patient outcomes, enabling healthcare professionals to make informed decisions in advance.

**The PROSPERO registration:**

CRD420251023018, https://www.crd.york.ac.uk/PROSPERO/view/CRD420251023018.

## 1. Introduction

Cardiac arrest (CA) represents a significant challenge to global health systems, posing a formidable threat to public health security [1, 2]. In a registry study focusing on out-of-hospital CA (OHCA), though spontaneous circulation is restored in 34.4% of patients, the prognosis for CA patients remains poor, with an overall survival rate of 9.6%. This situation imposes a notable burden on families and society [3]. The annual incidence of CA in the United States is 74.3 per 100,000 individuals, with a survival rate ranging from 10.8% to 11.4%. Notably, only 8.2% of patients achieve a favorable functional recovery. The overall incidence of CA in China is 97.1 per 100,000 population, and annually, approximately 1.03 million patients are afflicted with OHCA. However, the survival rate upon discharge is only 1.15% [4]. Therefore, finding simple and reliable prognostic predictive indicators is crucial for optimizing clinical decision-making and implementing individualized interventions.

Additionally, when discussing the incorporation of cardiopulmonary resuscitation into nursing objectives, clinicians rarely mention the prognostic factors and survival probabilities following CA [5]. Now, prognostic factors after CA are an important focus of ongoing research [6].

The red blood cell distribution width (RDW) reflects the heterogeneity in the volume of peripheral red blood cells. It is frequently used to identify hypoxemia and anemia [7]. Furthermore, the RDW level has been found to hold notable predictive value for mortality rates in critically ill patients [8]. Additionally, it is crucial in both short-term and long-term prognoses of various diseases, like vascular diseases, cardiovascular conditions, and peripheral artery diseases [9]. Some studies have found that elevated RDW levels are linked to adverse outcomes of CA [10, 11, 12, 13, 14, 15, 16, 17].

Thus, this study seeks to execute a systematic review and meta-analysis of current evidence regarding the connection of RDW with CA prognosis, clarify the strength and consistency, of its predictive power, and delve into the underlying causes of heterogeneity. The study results are expected to offer a low-cost, easily accessible prognostic assessment tool for clinical practice and shed new light on the role of RDW in the pathogenesis of CA.

## 2. Materials and Methods

The study was registered and implemented per the Preferred Reporting Items for Systematic Reviews and Meta-Analyses (PRISMA) guidelines extension for Diagnostic Test Accuracy (DTA) reviews [18], with a registration number of CRD420251023018.

### 2.1 Data Sources and Retrieval

The PubMed, Embase, Web of Science, and Cochrane databases were retrieved up to March 12, 2025. The search keywords included “cardiac arrest” and “red blood cell distribution width”, and were designed by combining medical subject headings and free-text terms via Boolean operators (AND, OR, NOT). In addition, the reference lists of relevant studies were examined to avoid missing any studies. Specific search strategies are available in **Supplementary Tables 1–4**.

### 2.2 Inclusion Criteria

All full-text articles in English with retrospective and prospective observational designs were included.

Inclusion criteria: (i) patients diagnosed with CA; (ii) RDW was detected; (iii) in-hospital mortality or survival was recorded during hospitalization or after discharge.

Exclusion criteria: (i) age <18 years; (ii) missing or uncollectible essential data from patients; (iii) irrelevant topics, letters, duplicate data or publications, reviews, meta-analyses, case reports, commentaries, editorials, or animal experiments, as well as studies that did not align with the research objectives and were not pertinent to the intervention or exposure factors.

### 2.3 Study Screening

Two investigators (YZ and BZ) independently screened the searched articles using EndNote. The titles and abstracts were read to exclude irrelevant studies. Full texts were then downloaded and read to determine eligible studies. Any disagreements were discussed with a third investigator (MY).

### 2.4 Data Extraction

Two investigators (YZ and BZ) independently extracted data from the included articles. The extracted data included first author, publication year, country, age, sex, standard mean deviation (SMD), adjusted hazard ratio (HR) and 95% confidence interval (CI), sensitivity (SEN), and specificity (SPE). Dissents, if any, were addressed by a third investigator (MY). In cases where the article did not provide SMD, the method proposed by Wan *et al*. [19] was employed to estimate the mean deviation.

### 2.5 Quality Assessment

Two investigators (YZ and BZ) independently employed the Newcastle-Ottawa scale (NOS) to assess the quality of the non-randomized trials [20]. The scale consists of eight questions divided into three domains: cohort selection, comparability, and outcome evaluation. Based on the relevant scoring criteria, a score of 1–3 represents low quality, while a score of 7–9 signifies high quality. Any disagreements were discussed with a third investigator (MY, QW, PS).

### 2.6 Statistical Analysis

The strength of the association of elevated RDW levels with adverse outcomes (in-hospital mortality) was measured by SMD and HR [21]. I^2^ statistics were used to measure heterogeneity. If I^2^ <50%, a fixed-effects model (FEM) was chosen. Otherwise, a random-effects model (REM) was used.

Subgroup analyses were performed by the following factors to identify sources of heterogeneity: region (Europe vs. Asia vs. Americas), study sample size (≥500 vs. <500), study type (retrospective study vs. prospective study), and time point (30-day mortality vs. not reported [NR] vs. 360-day mortality). Sensitivity analyses were conducted to ascertain the stability of the results.

Statistical analyses were carried out with STATA 15.0 (Company: StataCorp LLC; Location: College Station, TX; Country: USA), and the threshold effect was tested using MetaDisc (Team: Ramón y Cajal Hospital Team; Location: Madrid; Country: Spain). A *p*-value of less than 0.05 indicated statistical significance. A Fagan plot was employed to examine the prior and posterior probabilities. Funnel plots and Egger’s test were used to assess publication bias. An asymmetric funnel plot and a *p*-value < 0.05 from Egger’s test implied the existence of publication bias.

## 3. Results

### 3.1 Study Screening and Baseline Characteristics of the Included Studies

**Fig. 1.**
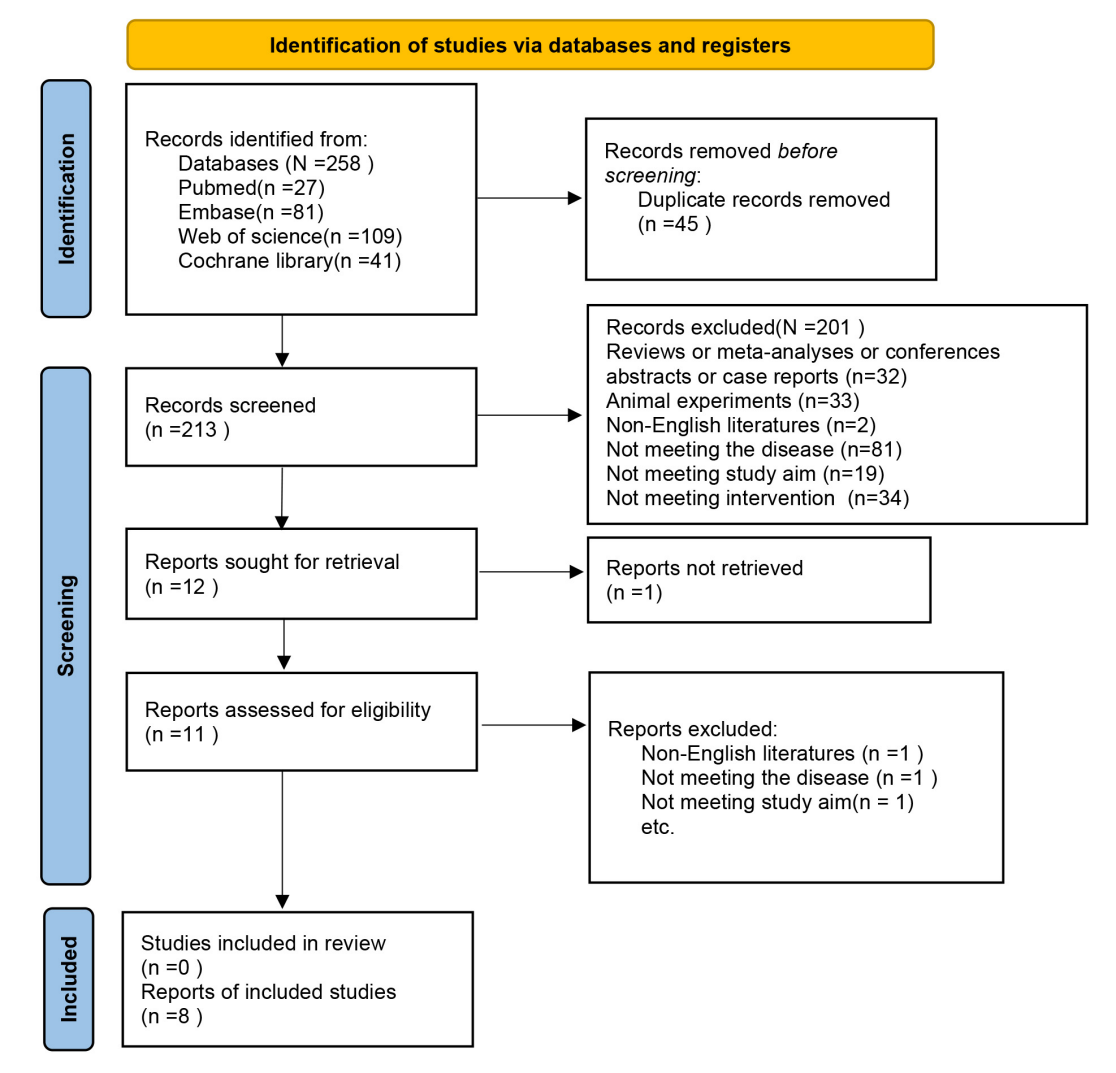
**Flow diagram of meta-analysis**.

A total of 258 records were collected from databases (Table 1, Ref. [10, 11, 12, 13, 14, 15, 16, 17]). After removing 45 duplicate publications, the titles and abstracts of 213 records were checked. Among these, 11 records were considered as potentially relevant, and their full-text articles were retrieved and reviewed. Following the full-text evaluation of the articles, three studies were excluded because of unrelated exposure factors and incompatible research objectives. In total, eight articles were included [10, 11, 12, 13, 14, 15, 16, 17]. The literature screening process is illustrated in Fig. 1.

**Table 1.** **Article baseline features**.

Id	Author	Year	Study design	Region	Participants	Sex ratio (M/F)	Age	Univariate/multivariate	AUC curve	Sensitivity	Specificity	Measurement time of RDW	The number of people with sepsis	Cut off
Associations between red cell distribution width and outcomes of adults with in-hospital cardiac arrest: a retrospective study	Yanwei Cheng *et al*. [10]	2022	Retrospective study	China	730	496/234	65.30 ± 16.34	Multivariate	0.64 (0.58–0.69)	78.60%	51.60%	On the day of ROSC (0 days)	15.30%	NR
Can red blood cell distribution width predict outcome after cardiac arrest?	Vito FONTANA *et al*. [11]	2018	Retrospective study	Belgium	390	275/115	66.36 ± 16.88	Univariate	0.60 (0.54–0.66)	71.00%	48.00%	Admission day (0 days) and once a day	30.76%	NR
Initial red cell distribution width as a predictor of poor neurological outcomes in out-of-hospital cardiac arrest survivors in a prospective, multicenter observational study (the KoCARC study)	Seon Hee Woo *et al*. [12]	2020	Prospective observational analysis	Korea	1008	717/291	61.90 ± 15.30	Multivariate	0.63 (0.59–0.67)	NR	NR	On the day of ROSC (0 days) (initial)	NR	NR
Red blood cell distribution width as an independent predictor of all-cause mortality in out-of-hospital cardiac arrest	Joonghee Kim *et al*. [13]	2012	Retrospective study	Korea	219	136/83	61.60 ± 20.60	Multivariate	0.61 (0.53–0.69) 30 d	NR	NR	On the day of ROSC (0 days)	NR	NR
									0.65 (0.57–0.72) 24 h					
Relationship between initial red cell distribution width and ΔRDW and mortality in cardiac arrest patients	Lei Zhong *et al*. [14]	2024	Retrospective study	MIMIC-IV database	1278	776/502	66.36 ± 16.87	Multivariate	30: development cohort: 0.72; validation cohort: 0.72.	NR	NR	At the admission of ROSC	NR	
									360: development cohort: 0.74; validation cohort: 0.73.					
The Relationship Between Hematological Parameters and Mortality in Cardiovascular Patients With Postcardiac Arrest Syndrome	Mehmet K Erol *et al*. [15]	2019	Retrospective study	Turkey	85	44/41	60.50 ± 18.90	Multivariate	NR	NR	NR	Initial blood sample at 0 h, 24 h, 48 h	NR	NR
The Association of Demographic Data and Hematological Parameters with Causes of Death in Patients Following Cardiopulmonary Resuscitation in the Emergency Department	Yunus Esen *et al*. [16]	2024	Retrospective study (case-control)	Turkey	292	182/112	58.81 ± 14.35	Univariate	0.67 (0.61–0.73)	86.00%	65.50%	NR	NR	14.47
Red blood cell distribution width for the prediction of outcomes after cardiac arrest	Tabita Urben *et al*. [17]	2023	Prospective observational analysis	Switzerland	702	506/196	64.70 ± 14.30	Multivariate	0.61	90.00%	15.20%	The average of RDW levels at Day 0, 1, 3, 5, 7 upon admission	NR	NR

AUC, area under the curve; RDW, red blood cell distribution width; ROSC, Return of spontaneous circulation; NR, not reported.

Eight eligible studies included 4702 patients. The patient was aged 58.65–66.3 years. There were two prospective studies [12, 17] and six retrospective studies [10, 11, 13, 14, 15, 16]. All studies reported in-hospital mortality. Three articles [12, 13, 14] reported 30-day mortality, one article [14] mentioned 360-day mortality, and four articles [10, 11, 16, 17] discussed SEN and SPE. The basic characteristics of the included studies are provided in Table 1.

### 3.2 Quality Assessment

All eight studies were rated as high-quality. The results of the risk of bias assessment are shown in Table 2 (Ref. [10, 11, 12, 13, 14, 15, 16, 17]).

**Table 2.** **Quality assessment results**.

Study	Selection				Comparability	Outcome			Quality scores
	Representativeness of the exposed cohort	Selection of the nonexposed cohort	Ascertainment of exposure	Demonstration that outcome of interest was not present at start of study	Comparability of cohorts on the basis of the design or analysis	Assessment of outcome	Was follow-up long enough for outcomes to occur	Adequacy of follow-up of cohorts	
Vito FONTANA *et al*. [11] 2018	✩	✩	✩	-	✩✩	✩	✩	✩	8
Yanwei Cheng *et al*. [10] 2022	✩	✩	✩	✩	✩✩	✩	✩	✩	9
Mehmet K. Erol *et al*. [15] 2019	✩	✩	✩	✩	✩	✩	✩	✩	8
Tabita Urben *et al*. [17] 2023	✩	✩	✩	✩	✩✩	✩	✩	✩	9
Yunus Esen *et al*. [16] 2024	✩	-	✩	✩	✩✩	✩	✩	✩	8
Lei Zhong *et al*. [14] 2024	✩	✩	✩	✩	✩✩	✩	✩	✩	9
Seon Hee Woo *et al*. [12] 2020	✩	-	✩	✩	✩✩	✩	✩	✩	8
Joonghee Kim *et al*. [13] 2012	✩	✩	✩	✩	✩✩	✩	✩	✩	9

Table Legend: The scoring system was based on the Newcastle-Ottawa Scale (NOS), where each “✩” corresponded to one point in the quality assessment of the included studies.

### 3.3 Meta-Analysis of the Association of RDW With the Prognosis of CA

#### 3.3.1 Differences in RDW Levels 

**Fig. 2.**
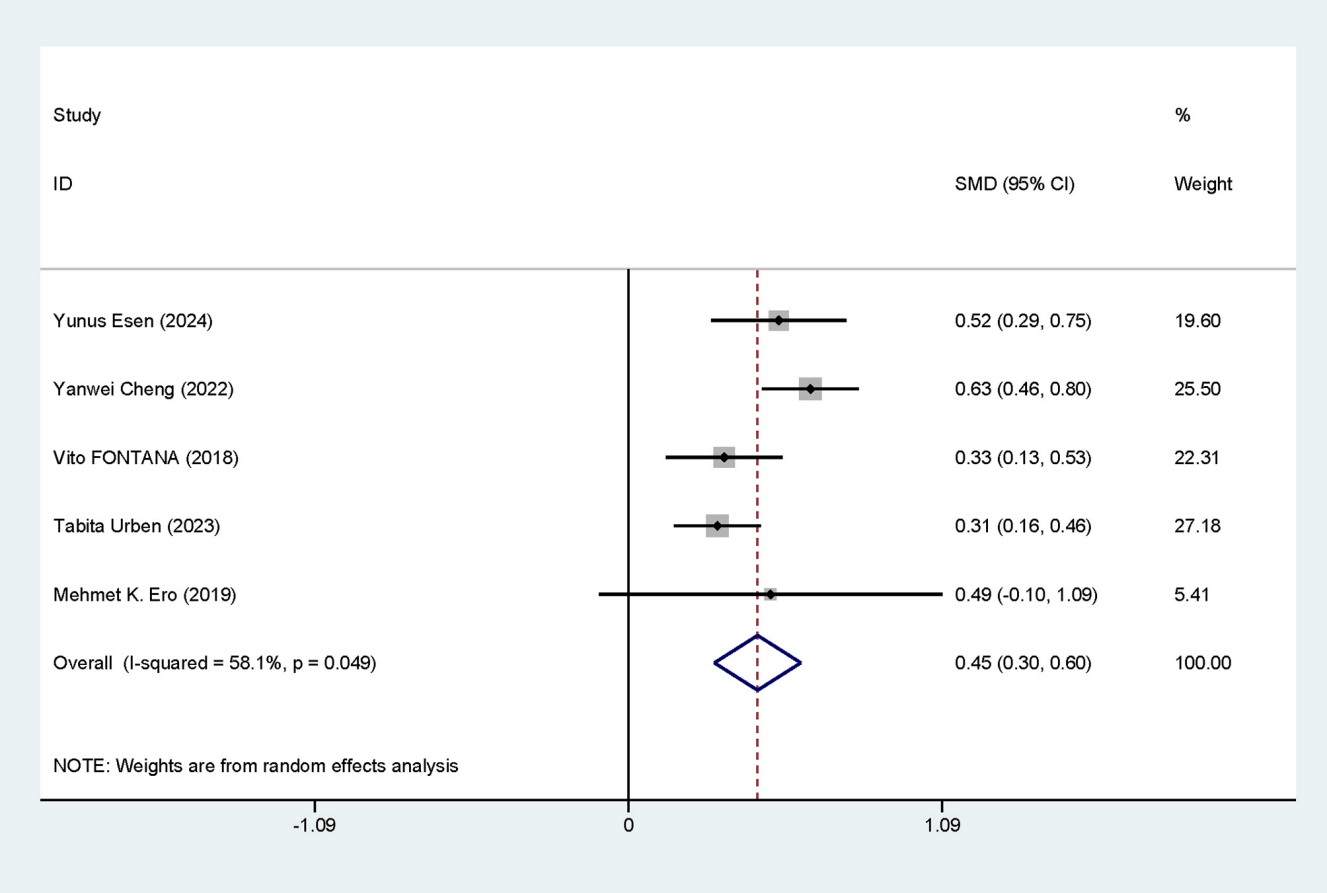
**Forest plot of RDW level differences**. SMD, standardized mean differences; CI, confidence intervals; RDW, red blood cell distribution width.

Five studies were included. Notable heterogeneity among studies was noted (I^2^ = 58.1%, *p* = 0.049). The data analysis was performed utilizing an REM. The results indicated that RDW levels were noticeably elevated in deceased patients after CA (SMD = 0.45, 95% CI: 0.30–0.60), with a statistically significant difference (*p* < 0.05) (Fig. 2).

#### 3.3.2 Subgroup Analysis

**Fig. 3.**
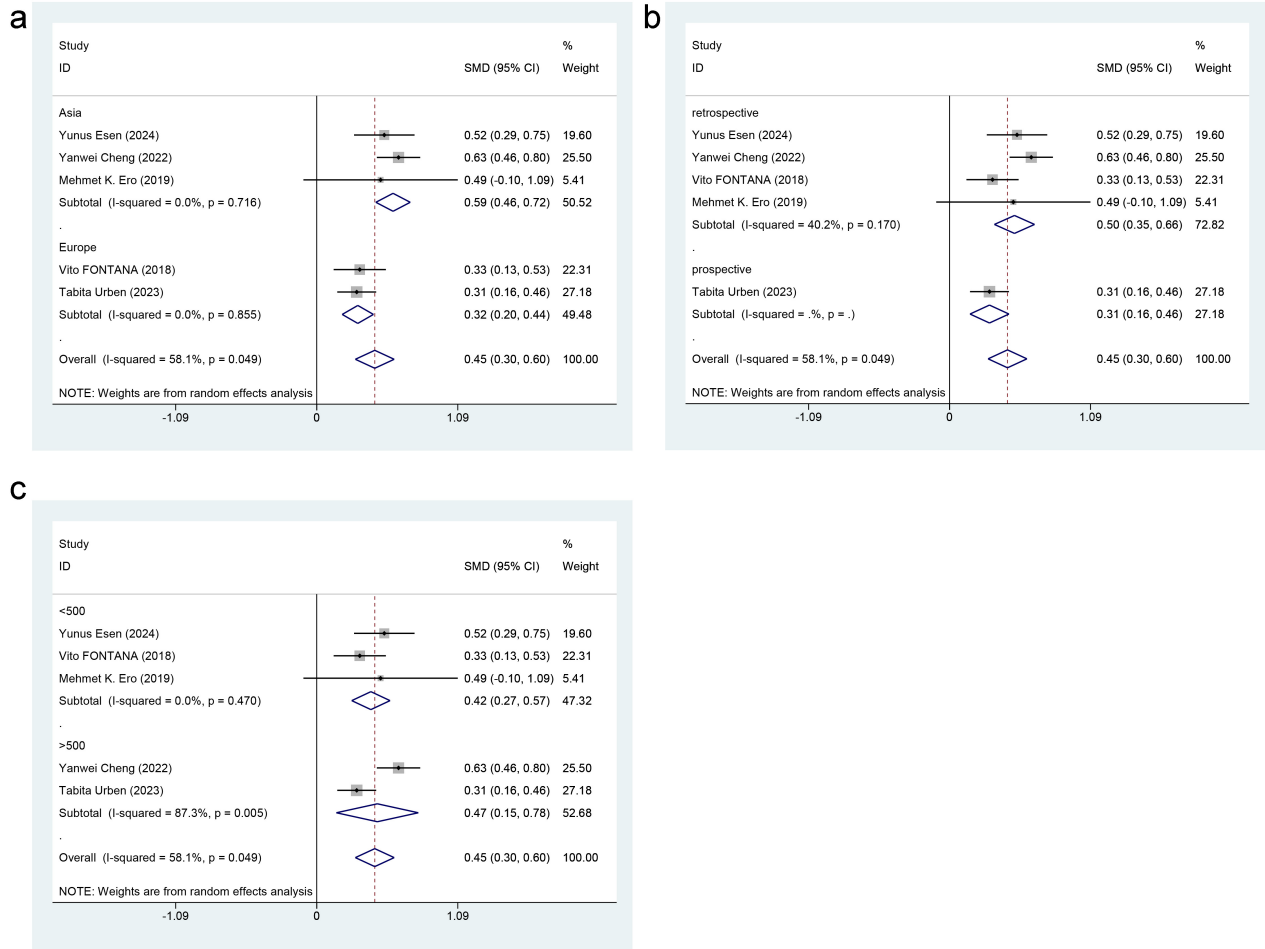
**Forest plot of subgroup analysis for RDW level differences**. (a) Region. (b) Study type. (c) Study sample size.

Subgroup analysis was performed to appraise whether region (Asia vs. Europe), study type (retrospective study vs. prospective study), and study sample size (≥500 vs. <500) contributed to the heterogeneity in RDW levels. The results demonstrated that region (Overall *p *= 0.049, I^2^ = 58.1%; Asia: SMD = 0.59, *p* = 0.716, I^2^ = 0.0%; Europe: SMD = 0.32, *p* = 0.855, I^2^ = 0.0%) and study type (Overall: *p* = 0.049, I^2^ = 58.1%; retrospective study: SMD = 0.50, *p* = 0.170, I^2^ = 40.2%; prospective study: SMD = 0.31, *p*-value was not displayed, I^2^ value was not displayed) might be sources of heterogeneity in the present findings. In contrast, the sample size was not a source of heterogeneity (Overall: *p* = 0.049, I^2^ = 58.1%; <500: SMD = 0.42, *p* = 0.470, I^2^ = 0.0%; ≥500: SMD = 0.47, *p* = 0.005, I^2^ = 87.3%) (Fig. 3).

#### 3.3.3 Mortality Risk

**Fig. 4.**
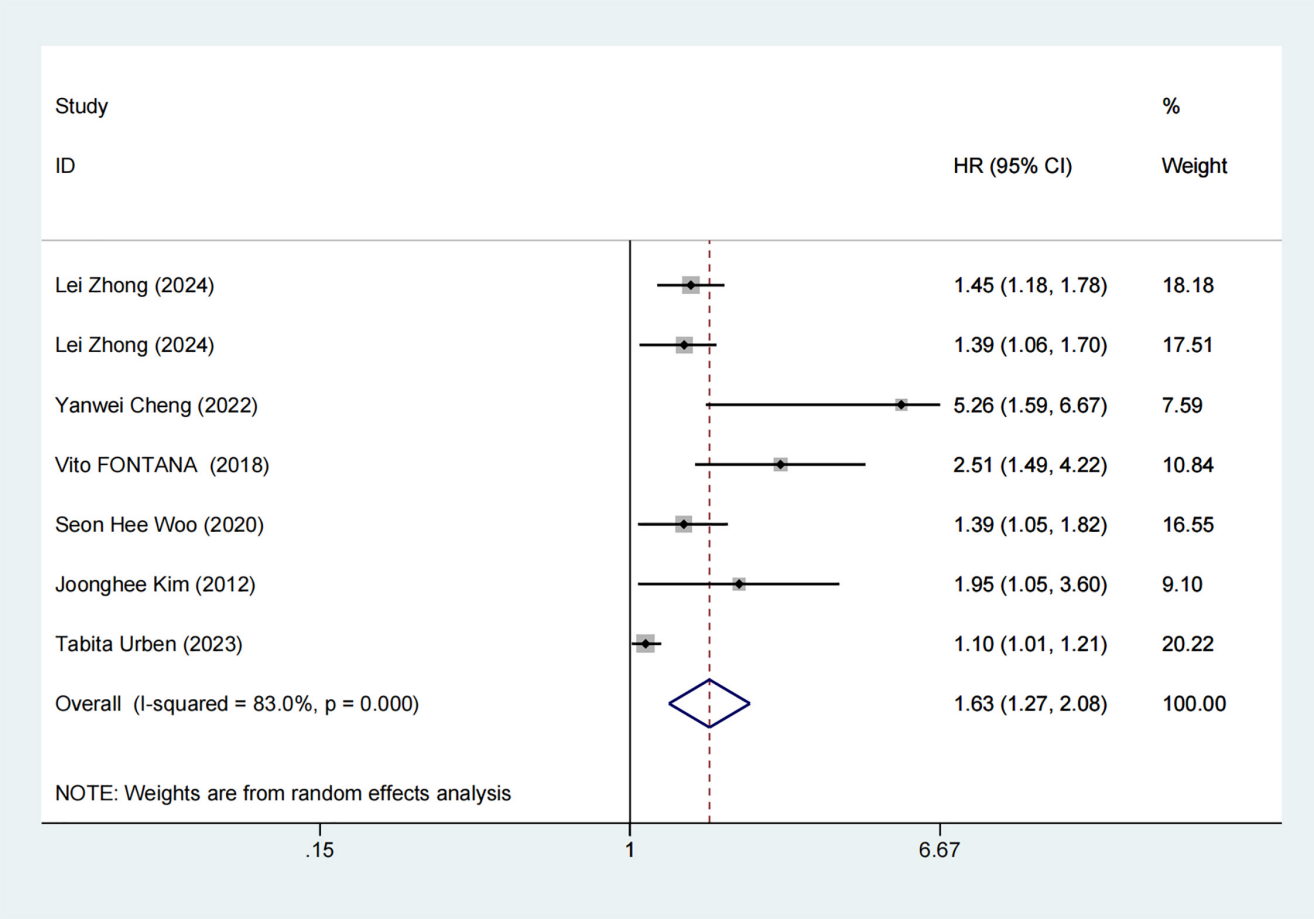
**Forest plot of mortality risk**. HR, hazard ratios.

Six articles reported mortality risks, with noticeable heterogeneity (I^2^ = 83%, *p* < 0.001). Thus, an REM was used. According to the meta-analysis, CA patients with elevated RDW levels exhibited a greater risk of mortality versus those with low RDW levels (HR = 1.63, 95% CI: 1.27–2.08) (Fig. 4).

#### 3.3.4 Subgroup Analysis

**Fig. 5.**
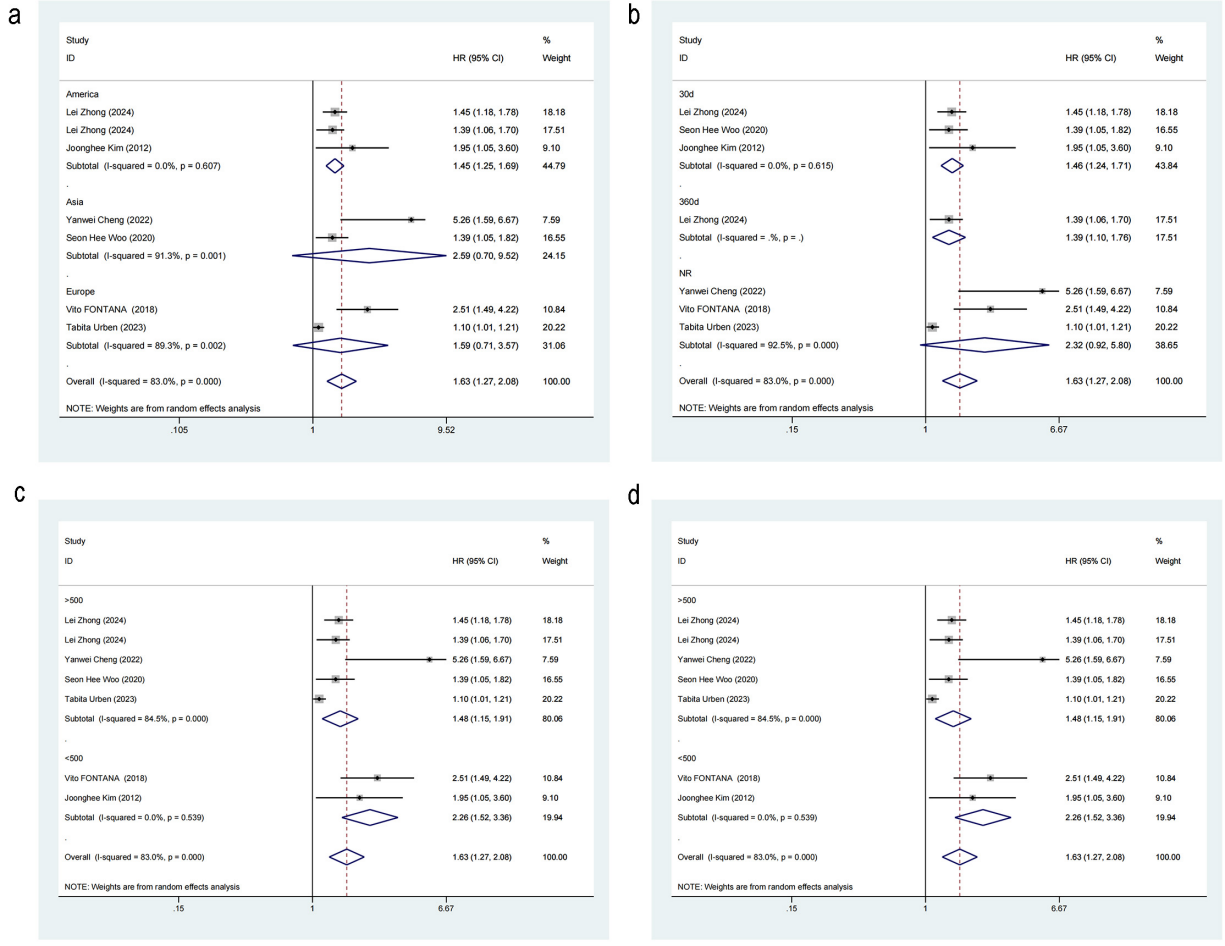
**Forest plot of subgroup analysis for mortality risk**. (a) Region. (b) Outcome time point. (c) Study sample size. (d) Study type.

Subgroup analysis was performed to ascertain whether region (Americas vs. Asia vs. Europe), study type (retrospective study vs. prospective study), sample size (≥500 vs. <500), and time point (30-day mortality vs. NR vs. 360-day mortality) contributed to the heterogeneity in the mortality risk. The results revealed that region (Overall: *p* < 0.001; Americas: HR = 1.45, 95% CI: 1.25–1.69, I^2^ = 83%, *p *= 0.607; Asia: HR = 2.59, 95% CI: 0.90–9.52, I^2^ = 91.3%, *p* < 0.001; Europe: HR = 1.59, 95% CI: 0.71–3.57, I^2^ = 91.3%, *p* = 0.002), sample size (Overall: *p* < 0.001; >500: HR = 1.48, 95% CI: 1.15–1.91, I^2^ = 84.5%, *p* < 0.001; <500: HR = 2.26, 95% CI: 1.52–3.36, I^2^ = 0.00%, *p* = 0.539), study type (Overall: *p* < 0.001; retrospective study: HR = 1.96, 95% CI: 1.39–2.75, I^2^ = 75.1%, *p* = 0.003; prospective study: HR = 1.96, 95% CI: 1.39–2.75, I^2^ = 60.2%, *p* = 0.113), time point (Overall: *p* < 0.001; 30-day mortality: HR = 1.46, 95% CI: 1.24–1.71, I^2^ = 0.0%, *p* = 0.615; NR: HR = 2.32, 95% CI: 0.92–5.80, I^2^ = 92.5%, *p* < 0.001, 360-day mortality: HR = 1.46, 95% CI: 1.24–1.71) were not sources of the heterogeneity (Fig. 5).

#### 3.3.5 Diagnostic Performance

The threshold effect was tested using MetaDisc, and the Spearman correlation was 0.2 between log(SEN) and log(1-SPE) (*p* = 0.800), implying no statistical significance. This result indicated there was no threshold effect.

**Fig. 6.**
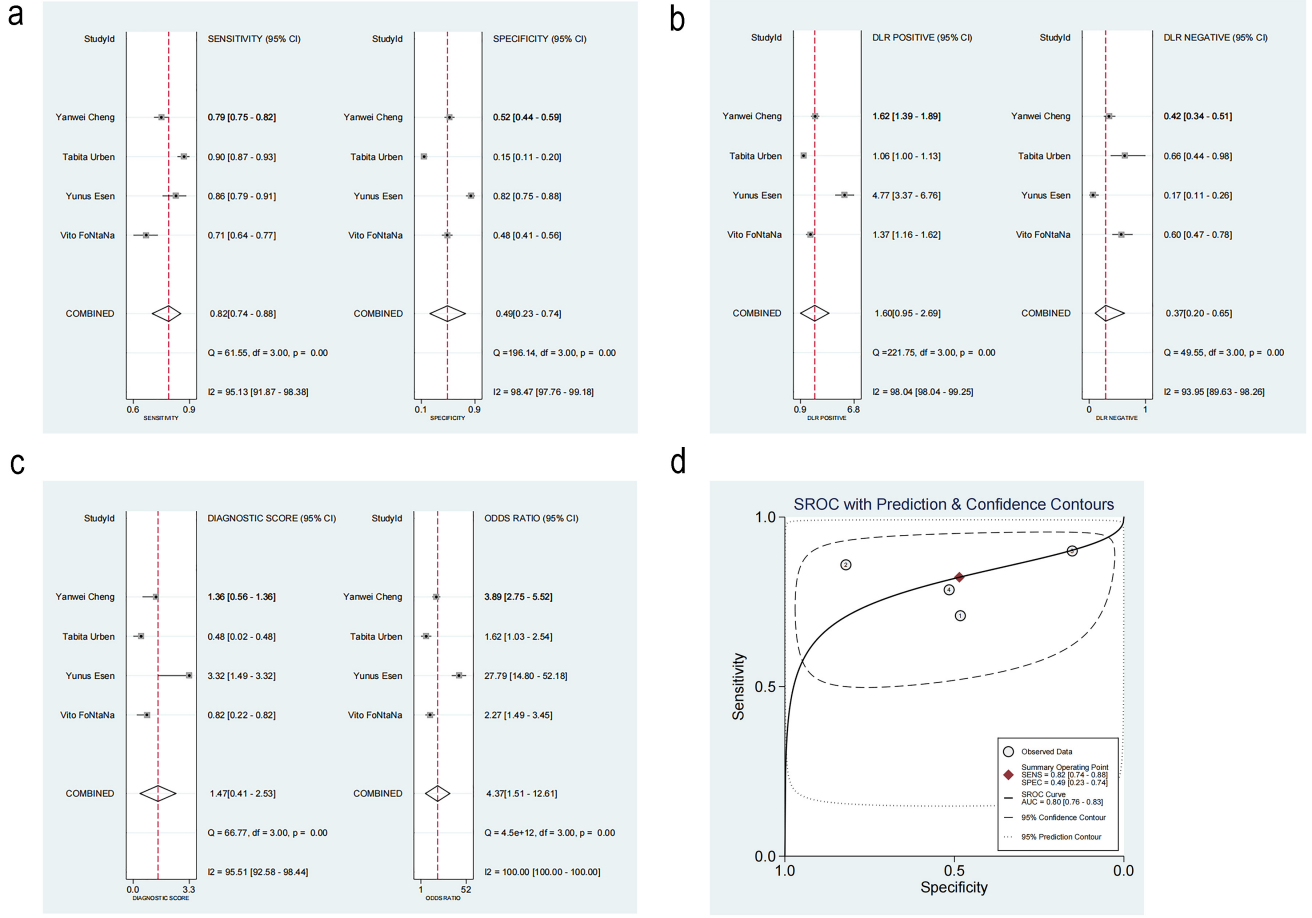
**Diagnosis analysis results**. (a) Forest plot of sensitivity and specificity. (b) Forest plot of likelihood ratios. (c) Forest plot of diagnostic performance. (d) Summary receiver operating characteristic curve. DLR, Design Layout Record; SROC, summary receiver operating characteristic. ①: Vito FONTANA [11]. 2018. ②: Yunus Esen [16]. 2024. ③: Tabita Urben [17]. 2023. ④: Yanwei Cheng [10]. 2022.

The pooled SEN and SPE were 0.82 (95% CI: 0.74–0.88, I^2^ = 95.13%) and 0.49 (95% CI: 0.23–0.74, I^2^ = 98.47%), respectively. Noticeable heterogeneity was detected (Fig. 6a).

The positive likelihood ratio (PLR) was 1.60 (95% CI: 0.95–2.69), while the negative likelihood ratio (NLR) was 0.37 (95% CI: 0.20–0.65) (Fig. 6b). The diagnostic odds ratio was 4.37 (95% CI: 1.51–12.61, I^2^ = 100%) (Fig. 6c).

**Fig. 7.**
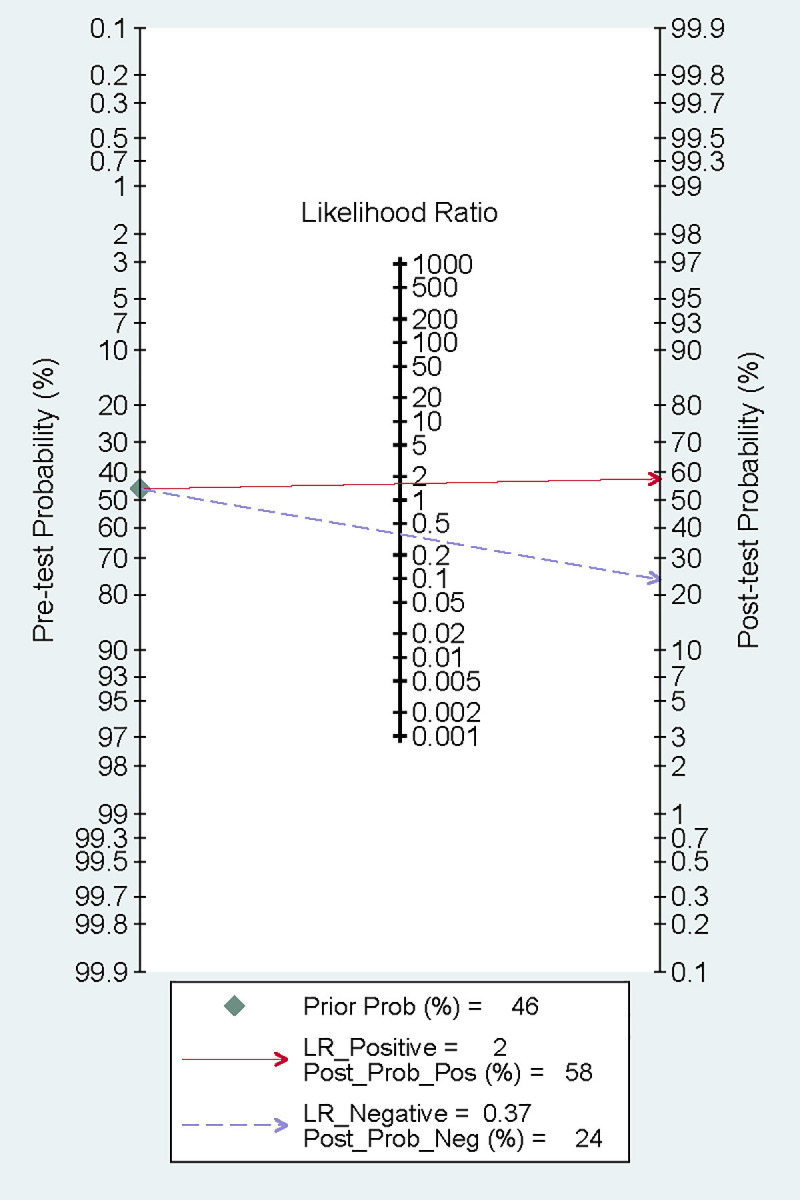
**Fagan plot**.

The summary receiver operating characteristic (SROC) curve is illustrated in Fig. 6d, Ref. [10, 11, 16, 17]. The area under the curve (AUC) was 0.80 (95% CI: 0.76–0.83), indicating satisfactory accuracy. A previous study reported that the mortality rate of patients with CA after resuscitation is as high as 46% [22]. According to the Fagan plot, with a PLR of 2, when the prior probability was 46%, the posterior probability was 58%. This detection method could enhance the probability of predicting mortality, thereby supporting its clinical applicability (Fig. 7).

#### 3.3.6 Sensitivity Analysis

**Fig. 8.**
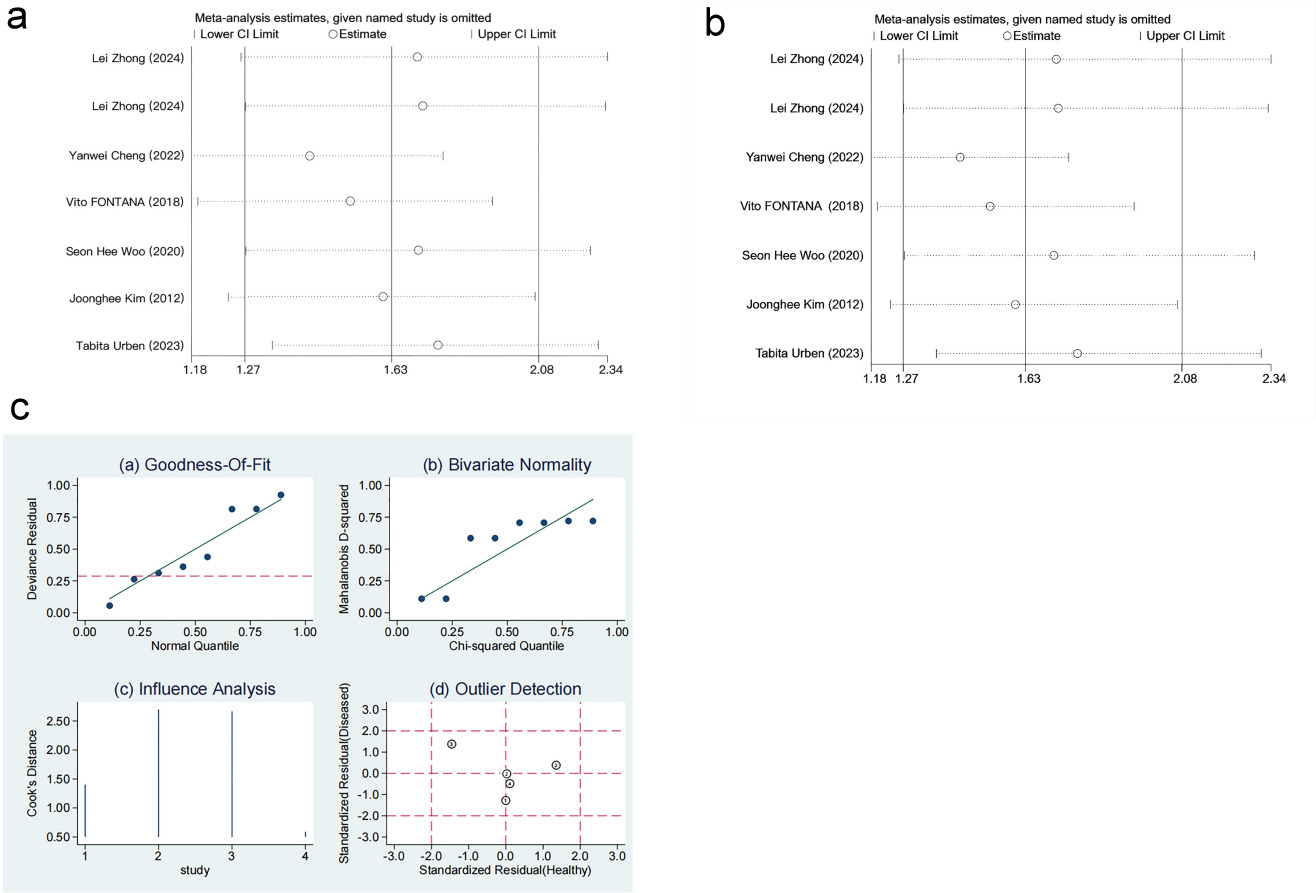
**Sensitivity analysis**. (a) Sensitivity analysis of level differences. (b) Sensitivity analysis of mortality risk. (c) Sensitivity analysis of diagnostic performance. Results from goodness-of-fit and bivariate normality analyses (Fig. 8c(a–b)) indicate that the bivariate model exhibits robustness. No outliers were detected via influence analysis and outlier detection (Fig. 8c(c–d)). ①: Vito FONTANA [11]. 2018. ②: Yunus Esen [16]. 2024. ③: Tabita Urben [17]. 2023. ④: Yanwei Cheng [10]. 2022.

The robustness of the results was verified by excluding each study individually. The sensitivity analysis demonstrated that the results were stable and reliable (Fig. 8a–c, Ref. [10, 11, 16, 17]).

#### 3.3.7 Publication Bias

**Fig. 9.**
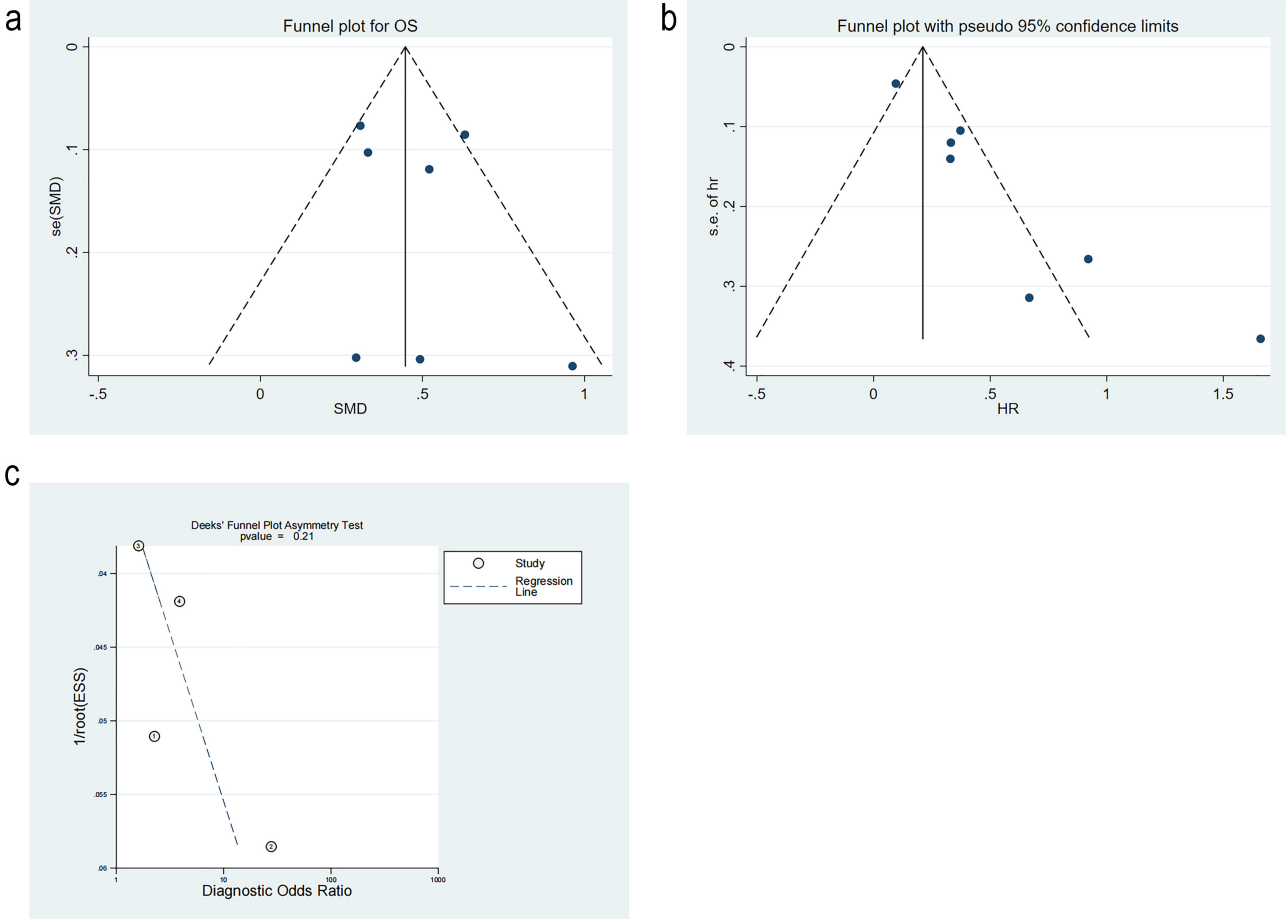
**Publication bias**. (a) Funnel plot of level difference. (b) Funnel plot of mortality risk. (c) Funnel plot of diagnostic performance. ①: Vito FONTANA [11]. 2018. ②: Yunus Esen [16]. 2024. ③: Tabita Urben [17]. 2023. ④: Yanwei Cheng [10]. 2022.

Studies on differences in RDW level were tested for publication bias by Egger’s test. The *p*-value was 0.61, and the funnel plot was symmetric, implying no publication bias. Studies on mortality risk were tested for publication bias. The funnel plot was significantly asymmetrical, indicating publication bias. The trim-and-fill method analysis estimated three missing studies on the left side of the funnel plot. The *p*-value was 0.001 before the trim-and-fill method, and 0.03 after the trim-and-fill method, indicating that the results remained statistically significant and were not reversed. This suggested that publication bias did not notably impact the findings. Studies on diagnostic performance were appraised for publication bias. The *p*-value from Deek’s test was 0.21, and the funnel plot was symmetrical, implying no publication bias (Fig. 9a–c, Ref. [10, 11, 16, 17]).

## 4. Discussion

This meta-analysis is the first to explore the prognostic significance of RDW in CA patients. The results reveal that high RDW levels are associated with a higher risk of mortality compared to low RDW levels in CA patients. Furthermore, the RDW levels in deceased CA patients are higher than those in survivors. These findings suggest that RDW is independently associated with patient outcomes and might be a valuable biomarker for prognosis.

The RDW data in the included articles are all obtained after baseline measurement of blood indicators of CA patients. The time of measurement is not specified accurately, and it reflects dynamic changes in the process. The articles do not mention whether the data are measured after the targeted body temperature management. Our meta-analysis results align with the findings reported by Tabita Urben *et al*. [17], indicating that RDW levels are associated with the prognosis in CA patients. This finding also aligns with the conclusion drawn by Mutlu *et al*. [23], which states that RDW levels increase on the day of CA. However, among the four studies reporting SEN and SPE [10, 11, 16, 17], the reported values of SEN and SPE vary significantly. Notably, the SPE reported in the study by Tabita Urben *et al*. [17] differs greatly from the other three studies. This difference may be attributable to the study population and sample size. Specifically, their study primarily involves an Asian cohort. Our findings indicate strong heterogeneity across studies from different regions. Additionally, differences in blood sample collection methods may contribute to these variations. The three studies collect samples during emergency situations, while their study obtains samples only after patients have survived long enough to be admitted to the Intensive Care Unit (ICU). Only one of the eight articles mentions the cutoff value, and we speculate that the cutoff values of the eight articles may be different [10, 11, 12, 13, 14, 16, 17], possibly due to different analytical techniques used and inconsistent results [24, 25]. Some included studies report different HR and SMD values, likely due to the initial conditions of the patients. RDW levels might vary with the initial clinical status of OHCA patients. For example, in a meta-analysis, there is a certain association of RDW levels with sepsis [26]. More than one-third of patients after CA may also develop bacteremia [27]. Therefore, it is necessary to further exclude patients with sepsis among those diagnosed with CA in order to ascertain the prognostic significance of RDW for this patient population.

The mechanism by which elevated RDW levels in CA patients are linked to poor prognosis remains unclear. RDW is associated with increased levels of B-type natriuretic peptide and poor cardiac contractility [28, 29], which might be due to the deformability of red blood cells. Changes in osmotic pressure would affect this deformity and the microvascular blood flow, eventually manifesting as cardiovascular disease [30]. It may also be specifically related to certain pathophysiological factors of CA syndrome. CA can induce ischemia-reperfusion injury to the myocardium. This injury may result in cellular apoptosis and tissue dysfunction via different physiological mechanisms, like inflammatory responses, reactive oxygen species, mitochondrial dysfunction, and impaired protein synthesis [31, 32]. Elevated RDW levels have been clearly associated with inflammation and oxidative stress [33, 34]. Therefore, we hypothesize that the elevated RDW levels in CA patients are attributable to a systemic inflammatory response. Pro-inflammatory cytokines have been proven to restrict the maturation and proliferation of erythropoietin-induced erythrocytes, as well as decrease the expression of erythropoietin receptors related to increased RDW levels. Oxidative stress diminishes the survival of red blood cells and impacts bone marrow function; premature red blood cells contribute to a rise in RDW levels and red blood cells released in peripheral circulation [35, 36, 37]. CA patients also have myocardial dysfunction. An activated renin-angiotensin-aldosterone system can cause an increase in RDW levels [38]. These patients may often be afflicted with hypoxemia. The increase in erythropoietin levels due to hypoxia might raise RDW levels, subsequently impairing the deformability and survival rate of red blood cells. Reduced deformability of red blood cells can compromise cerebral microcirculation, potentially causing tissue hypoxia and increasing the risk of poor prognosis for patients [39]. Elevated RDW levels directly reflect the cholesterol content in red blood cell membranes. It is a widely recognized risk factor for acute coronary syndrome and CA [40].

This meta-analysis has several limitations. First, the number of studies and patients included in this analysis is limited, and it only encompasses observational studies. Consequently, the evidence chain presented in this study may not be robust enough. Additionally, we have solely utilized patient survival as an outcome measure [41]. However, the relationship between this exposure factor and neurological prognosis at discharge or long-term survival remains unknown. Thus, further similar studies are necessary to enhance our understanding and provide better guidance for clinical practitioners and nursing staff. Second, there is heterogeneity in our study. The subgroup analysis for mortality risk indicates that time points may be a potential source of heterogeneity, while the subgroup analysis on differences in RDW levels indicates study type as a potential source of heterogeneity. To address the heterogeneity, it is essential to conduct more detailed and comprehensive further research. Third, although we have used SMDs and an REM to determine the strength of the link between elevated RDW levels and adverse outcomes, the publication bias in the meta-analysis concerning mortality risk remains inadequately addressed.

RDW is a routine and cost-effective hematological marker in clinical practice. Our meta-analysis demonstrates a significant association of RDW with CA patients, suggesting its potential utility in the early management of these patients. Furthermore, integrating RDW with established clinical models, such as the PROLOGUE score, may enhance its prognostic performance. Compared with other indicators for predicting the prognosis of CA patients, RDW, as an indicator that reflects blood changes, can simultaneously reflect various pathological processes that damage the body after CA, including oxidative stress, inflammatory response, and malnutrition. It is relatively independent of diseases related to the cardiac system and is not easily affected by other disease factors and other interferences. Hu *et al*. [42] found an association between elevated RDW and mortality in 412 cardiac intensive care unit (CICU) patients. Even after adjusting for disease severity and other variables, higher RDW was still associated with lower long-term survival [42]. Bazick *et al*. [43] reported a strong independent relationship between RDW and mortality in a large study of RDW in critically ill patients. Some other conventional indicators, such as lactate, have been shown to be prognostic factors for CA patients. However, some studies have pointed out that this indicator is easily affected by other metabolites and undetected weak acids, resulting in negative results [44]. However, elevated RDW levels can be influenced by various factors, including age, race, pregnancy status, renal disease, and sepsis [44, 45]. RDW was indeed confirmed to be associated with sepsis in a meta-analysis [46]. Nevertheless, only two of the articles included in our analysis reported data on sepsis (only the percentage of sepsis) [10, 11]. This does not indicate whether sepsis is a complication or a cause of CA. Moreover, the clinical diagnosis of sepsis concurrent with CA is relatively difficult because both can lead to systemic organ failure. In addition, patients after cardiopulmonary resuscitation may also show symptoms similar to sepsis, such as increased plasma cytokines and endotoxins [46]. Therefore, we believe that sepsis has relatively few confounding factors in this meta-analysis.

Currently, it remains unclear how to adjust the measurement methods of RDW based on physiological factors such as age. Future prospective studies are required to elucidate this issue further. Additionally, the pathological mechanisms underlying elevated RDW levels in CA patients need to be further explored.

## 5. Conclusion

RDW levels in CA patients are associated with poor outcomes. According to our findings, RDW levels might be a valuable marker of prognosis in CA patients. However, related research still needs to include more samples and different types of studies to further validate these findings. At the same time, some related factors that may also affect RDW levels, such as sepsis, need to be excluded.

## Availability of Data and Materials

All data generated or analysed during this study are included in this published article and its **Supplementary information files**.

## Supplementary Material

Supplementary material associated with this article can be found, in the online version, at https://doi.org/10.31083/RCM43774.
