# Supplementary material for: Predictive Value of Red Blood Cell Distribution Width for the Prognosis of Cardiac Arrest: A Systematic Review and Meta-Analysis
Source: Rev Cardiovasc Med. 2025 Nov 17;26(11):43774. doi: 10.31083/RCM43774 (PMC12680998; doi:10.31083/RCM43774)
Supplement: Supplementary file 1 [file 2153-8174-26-11-43774-s1.zip › Supplementary Material.docx]

Supplementary Table 1. Search Strategies for PubMed.

| Pubmed | | |
| --- | --- | --- |
| # | Query | Results |
| 1 | Heart Arrest[MeSH Terms] | 59263 |
| 2 | “asystol*”[Title/Abstract] OR “cardiac arrest”[Title/Abstract] OR “Cardiopulmonary Arrest”[Title/Abstract] OR “circulation arrest”[Title/Abstract] OR “circulatory arrest”[Title/Abstract] OR “heart arrest”[Title/Abstract] OR “heart asystole”[Title/Abstract] OR “heart standstill”[Title/Abstract] | 62166 |
| 3 | Heart Arrest[MeSH Terms] OR “asystol*”[Title/Abstract] OR “cardiac arrest”[Title/Abstract] OR “Cardiopulmonary Arrest”[Title/Abstract] OR “circulation arrest”[Title/Abstract] OR “circulatory arrest”[Title/Abstract] OR “heart arrest”[Title/Abstract] OR “heart asystole”[Title/Abstract] OR “heart standstill”[Title/Abstract] | 90552 |
| 4 | Erythrocyte Indices[MeSH Terms] | 6612 |
| 5 | “Erythrocyte Diameter*”[Title/Abstract] OR “erythrocyte distribution width”[Title/Abstract] OR “Erythrocyte Index*”[Title/Abstract] OR “Erythrocyte Indices”[Title/Abstract] OR “Erythrocyte Size Determination*”[Title/Abstract] OR “Erythrocyte Thickness”[Title/Abstract] OR “Mean Cell Hemoglobin Concentration”[Title/Abstract] OR “Mean Cell Volume*”[Title/Abstract] OR “Mean Corpuscular Hemoglobin*”[Title/Abstract] OR “Mean Corpuscular Hemoglobulin Concentration”[Title/Abstract] OR “Mean Corpuscular Volume*”[Title/Abstract] OR “RCDW”[Title/Abstract] OR “RDW”[Title/Abstract] OR “red blood cell distribution width”[Title/Abstract] OR “Red Cell Distribution Width”[Title/Abstract] OR “Red Cell Index*”[Title/Abstract] OR “Red Cell Indices”[Title/Abstract] | 12405 |
| 6 | Erythrocyte Indices[MeSH Terms] OR “Erythrocyte Diameter*”[Title/Abstract] OR “erythrocyte distribution width”[Title/Abstract] OR “Erythrocyte Index*”[Title/Abstract] OR “Erythrocyte Indices”[Title/Abstract] OR “Erythrocyte Size Determination*”[Title/Abstract] OR “Erythrocyte Thickness”[Title/Abstract] OR “Mean Cell Hemoglobin Concentration”[Title/Abstract] OR “Mean Cell Volume*”[Title/Abstract] OR “Mean Corpuscular Hemoglobin*”[Title/Abstract] OR “Mean Corpuscular Hemoglobulin Concentration”[Title/Abstract] OR “Mean Corpuscular Volume*”[Title/Abstract] OR “RCDW”[Title/Abstract] OR “RDW”[Title/Abstract] OR “red blood cell distribution width”[Title/Abstract] OR “Red Cell Distribution Width”[Title/Abstract] OR “Red Cell Index*”[Title/Abstract] OR “Red Cell Indices”[Title/Abstract] | 15308 |
| 7 | Heart Arrest[MeSH Terms] OR “asystol*”[Title/Abstract] OR “cardiac arrest”[Title/Abstract] OR “Cardiopulmonary Arrest”[Title/Abstract] OR “circulation arrest”[Title/Abstract] OR “circulatory arrest”[Title/Abstract] OR “heart arrest”[Title/Abstract] OR “heart asystole”[Title/Abstract] OR “heart standstill”[Title/Abstract] AND Erythrocyte Indices[MeSH Terms] OR “Erythrocyte Diameter*”[Title/Abstract] OR “erythrocyte distribution width”[Title/Abstract] OR “Erythrocyte Index*”[Title/Abstract] OR “Erythrocyte Indices”[Title/Abstract] OR “Erythrocyte Size Determination*”[Title/Abstract] OR “Erythrocyte Thickness”[Title/Abstract] OR “Mean Cell Hemoglobin Concentration”[Title/Abstract] OR “Mean Cell Volume*”[Title/Abstract] OR “Mean Corpuscular Hemoglobin*”[Title/Abstract] OR “Mean Corpuscular Hemoglobulin Concentration”[Title/Abstract] OR “Mean Corpuscular Volume*”[Title/Abstract] OR “RCDW”[Title/Abstract] OR “RDW”[Title/Abstract] OR “red blood cell distribution width”[Title/Abstract] OR “Red Cell Distribution Width”[Title/Abstract] OR “Red Cell Index*”[Title/Abstract] OR “Red Cell Indices”[Title/Abstract] | 27 |

Supplementary Table 2. Search Strategies for Embase.

| Embase | | |
| --- | --- | --- |
| # | Query | Results |
| 1 | ‘heart arrest’/exp | 150015 |
| 2 | ‘asystol*’:ti,ab,kw OR ‘cardiac arrest’:ti,ab,kw OR ‘cardiopulmonary arrest’:ti,ab,kw OR ‘circulation arrest’:ti,ab,kw OR ‘circulatory arrest’:ti,ab,kw OR ‘heart arrest’:ti,ab,kw OR ‘heart asystole’:ti,ab,kw OR ‘heart standstill’:ti,ab,kw | 100987 |
| 3 | ‘heart arrest’/exp OR ‘asystol*’:ti,ab,kw OR ‘cardiac arrest’:ti,ab,kw OR ‘cardiopulmonary arrest’:ti,ab,kw OR ‘circulation arrest’:ti,ab,kw OR ‘circulatory arrest’:ti,ab,kw OR ‘heart arrest’:ti,ab,kw OR ‘heart asystole’:ti,ab,kw OR ‘heart standstill’:ti,ab,kw | 168046 |
| 4 | ‘red blood cell distribution width’/exp | 7103 |
| 5 | ‘erythrocyte diameter*’:ti,ab,kw OR ‘erythrocyte distribution width’:ti,ab,kw OR ‘erythrocyte index*’:ti,ab,kw OR ‘erythrocyte indices’:ti,ab,kw OR ‘erythrocyte size determination*’:ti,ab,kw OR ‘erythrocyte thickness’:ti,ab,kw OR ‘mean cell hemoglobin concentration’:ti,ab,kw OR ‘mean cell volume*’:ti,ab,kw OR ‘mean corpuscular hemoglobin*’:ti,ab,kw OR ‘mean corpuscular hemoglobulin concentration’:ti,ab,kw OR ‘mean corpuscular volume*’:ti,ab,kw OR ‘rcdw’:ti,ab,kw OR ‘rdw’:ti,ab,kw OR ‘red blood cell distribution width’:ti,ab,kw OR ‘red cell distribution width’:ti,ab,kw OR ‘red cell index*’:ti,ab,kw OR ‘red cell indices’:ti,ab,kw | 18776 |
| 6 | ‘red blood cell distribution width’/exp OR ‘erythrocyte diameter*’:ti,ab,kw OR ‘erythrocyte distribution width’:ti,ab,kw OR ‘erythrocyte index*’:ti,ab,kw OR ‘erythrocyte indices’:ti,ab,kw OR ‘erythrocyte size determination*’:ti,ab,kw OR ‘erythrocyte thickness’:ti,ab,kw OR ‘mean cell hemoglobin concentration’:ti,ab,kw OR ‘mean cell volume*’:ti,ab,kw OR ‘mean corpuscular hemoglobin*’:ti,ab,kw OR ‘mean corpuscular hemoglobulin concentration’:ti,ab,kw OR ‘mean corpuscular volume*’:ti,ab,kw OR ‘rcdw’:ti,ab,kw OR ‘rdw’:ti,ab,kw OR ‘red blood cell distribution width’:ti,ab,kw OR ‘red cell distribution width’:ti,ab,kw OR ‘red cell index*’:ti,ab,kw OR ‘red cell indices’:ti,ab,kw | 21807 |
| 7 | ‘heart arrest’/exp OR ‘asystol*’:ti,ab,kw OR ‘cardiac arrest’:ti,ab,kw OR ‘cardiopulmonary arrest’:ti,ab,kw OR ‘circulation arrest’:ti,ab,kw OR ‘circulatory arrest’:ti,ab,kw OR ‘heart arrest’:ti,ab,kw OR ‘heart asystole’:ti,ab,kw OR ‘heart standstill’:ti,ab,kw AND ‘red blood cell distribution width’/exp OR ‘erythrocyte diameter*’:ti,ab,kw OR ‘erythrocyte distribution width’:ti,ab,kw OR ‘erythrocyte index*’:ti,ab,kw OR ‘erythrocyte indices’:ti,ab,kw OR ‘erythrocyte size determination*’:ti,ab,kw OR ‘erythrocyte thickness’:ti,ab,kw OR ‘mean cell hemoglobin concentration’:ti,ab,kw OR ‘mean cell volume*’:ti,ab,kw OR ‘mean corpuscular hemoglobin*’:ti,ab,kw OR ‘mean corpuscular hemoglobulin concentration’:ti,ab,kw OR ‘mean corpuscular volume*’:ti,ab,kw OR ‘rcdw’:ti,ab,kw OR ‘rdw’:ti,ab,kw OR ‘red blood cell distribution width’:ti,ab,kw OR ‘red cell distribution width’:ti,ab,kw OR ‘red cell index*’:ti,ab,kw OR ‘red cell indices’:ti,ab,kw | 81 |

Supplementary Table 3. Search Strategies for the Cochrane Library.

| Cochrane Library | | |
| --- | --- | --- |
| # | Query | Results |
| 1 | MeSH descriptor: [Heart Arrest] explode all trees | 3075 |
| 2 | (‘asystol*’ OR ‘cardiac arrest’ OR ‘Cardiopulmonary Arrest’ OR ‘circulation arrest’ OR ‘circulatory arrest’ OR ‘heart arrest’ OR ‘heart asystole’ OR ‘heart standstill’):ti,ab,kw | 7480 |
| 3 | MeSH descriptor: [Heart Arrest] explode all trees OR (‘asystol*’ OR ‘cardiac arrest’ OR ‘Cardiopulmonary Arrest’ OR ‘circulation arrest’ OR ‘circulatory arrest’ OR ‘heart arrest’ OR ‘heart asystole’ OR ‘heart standstill’):ti,ab,kw | 8289 |
| 4 | MeSH descriptor: [Erythrocyte Indices] explode all trees | 222 |
| 5 | (‘Erythrocyte Diameter*’ OR ‘erythrocyte distribution width’ OR ‘Erythrocyte Index*’ OR ‘Erythrocyte Indices’ OR ‘Erythrocyte Size Determination*’ OR ‘Erythrocyte Thickness’ OR ‘Mean Cell Hemoglobin Concentration’ OR ‘Mean Cell Volume*’ OR ‘Mean Corpuscular Hemoglobin*’ OR ‘Mean Corpuscular Hemoglobulin Concentration’ OR ‘Mean Corpuscular Volume*’ OR ‘RCDW’ OR ‘RDW’ OR ‘red blood cell distribution width’ OR ‘Red Cell Distribution Width’ OR ‘Red Cell Index*’ OR ‘Red Cell Indices’):ti,ab,kw | 6769 |
| 6 | MeSH descriptor: [Erythrocyte Indices] explode all trees OR (‘Erythrocyte Diameter*’ OR ‘erythrocyte distribution width’ OR ‘Erythrocyte Index*’ OR ‘Erythrocyte Indices’ OR ‘Erythrocyte Size Determination*’ OR ‘Erythrocyte Thickness’ OR ‘Mean Cell Hemoglobin Concentration’ OR ‘Mean Cell Volume*’ OR ‘Mean Corpuscular Hemoglobin*’ OR ‘Mean Corpuscular Hemoglobulin Concentration’ OR ‘Mean Corpuscular Volume*’ OR ‘RCDW’ OR ‘RDW’ OR ‘red blood cell distribution width’ OR ‘Red Cell Distribution Width’ OR ‘Red Cell Index*’ OR ‘Red Cell Indices’):ti,ab,kw | 6769 |
| 7 | MeSH descriptor: [Heart Arrest] explode all trees OR (‘asystol*’ OR ‘cardiac arrest’ OR ‘Cardiopulmonary Arrest’ OR ‘circulation arrest’ OR ‘circulatory arrest’ OR ‘heart arrest’ OR ‘heart asystole’ OR ‘heart standstill’):ti,ab,kw AND MeSH descriptor: [Erythrocyte Indices] explode all trees OR (‘Erythrocyte Diameter*’ OR ‘erythrocyte distribution width’ OR ‘Erythrocyte Index*’ OR ‘Erythrocyte Indices’ OR ‘Erythrocyte Size Determination*’ OR ‘Erythrocyte Thickness’ OR ‘Mean Cell Hemoglobin Concentration’ OR ‘Mean Cell Volume*’ OR ‘Mean Corpuscular Hemoglobin*’ OR ‘Mean Corpuscular Hemoglobulin Concentration’ OR ‘Mean Corpuscular Volume*’ OR ‘RCDW’ OR ‘RDW’ OR ‘red blood cell distribution width’ OR ‘Red Cell Distribution | 41 |

Supplementary Table 4. Search Strategies for Web of Science.

| Web of Science | | |
| --- | --- | --- |
| # | Query | Results |
| 1 | TS=((asystol*) OR (cardiac arrest) OR (Cardiopulmonary Arrest) OR (circulation arrest) OR (circulatory arrest) OR (heart arrest) OR (heart asystole) OR (heart standstill)) | 64496 |
| 2 | TS=((Erythrocyte Diameter*) OR (erythrocyte distribution width) OR (Erythrocyte Index*) OR (Erythrocyte Indices) OR (Erythrocyte Size Determination*) OR (Erythrocyte Thickness) OR (Mean Cell Hemoglobin Concentration) OR (Mean Cell Volume*) OR (Mean Corpuscular Hemoglobin*) OR (Mean Corpuscular Hemoglobulin Concentration) OR (Mean Corpuscular Volume*) OR (RCDW) OR (RDW) OR (red blood cell distribution width) OR (Red Cell Distribution Width) OR (Red Cell Index*) OR (Red Cell Indices)) | 48340 |
| 3 | TS=((asystol*) OR (cardiac arrest) OR (Cardiopulmonary Arrest) OR (circulation arrest) OR (circulatory arrest) OR (heart arrest) OR (heart asystole) OR (heart standstill)) AND TS=((Erythrocyte Diameter*) OR (erythrocyte distribution width) OR (Erythrocyte Index*) OR (Erythrocyte Indices) OR (Erythrocyte Size Determination*) OR (Erythrocyte Thickness) OR (Mean Cell Hemoglobin Concentration) OR (Mean Cell Volume*) OR (Mean Corpuscular Hemoglobin*) OR (Mean Corpuscular Hemoglobulin Concentration) OR (Mean Corpuscular Volume*) OR (RCDW) OR (RDW) OR (red blood cell distribution width) OR (Red Cell Distribution Width) OR (Red Cell Index*) OR (Red Cell Indices)) | 109 |
